# Profiles of 14-3-3 and Total Tau in CSF Samples of Chinese Patients of Different Genetic Prion Diseases

**DOI:** 10.3389/fnins.2019.00934

**Published:** 2019-09-04

**Authors:** Cao Chen, Chao Hu, Qi Shi, Wei Zhou, Kang Xiao, Yuan Wang, Lian Liu, Jia Chen, Ying Xia, Xiao-Ping Dong

**Affiliations:** ^1^State Key Laboratory of Infectious Disease Prevention and Control, NHC Key Laboratory of Medical Virology and Viral Diseases, National Institute for Viral Disease Control and Prevention, Chinese Center for Disease Control and Prevention, Beijing, China; ^2^Center for Biosafety Mega-Science, Chinese Academy of Sciences, Wuhan, China; ^3^College of Life Science and Technology, Heilongjiang Bayi Agricultural University, Daqing, China; ^4^Collaborative Innovation Center for Diagnosis and Treatment of Infectious Diseases, Zhejiang University, Hangzhou, China; ^5^Center for Global Public Health, Chinese Center for Disease Control and Prevention, Beijing, China; ^6^China Academy of Chinese Medical Sciences, Beijing, China

**Keywords:** prion, genetic prion disease, CSF, 14-3-3, tau

## Abstract

**Background:**

The abnormal alterations of proteins 14-3-3 and tau in cerebrospinal fluid (CSF) are widely used for the diagnosis of sporadic Creutzfeldt-Jakob disease (sCJD), while the situations of CSF biomarkers in genetic prion diseases (gPrDs), particularly in Chinese gPrDs patients, have not been well documented.

**Methods:**

Here, with the help of commercial 14-3-3 and total tau ELISA kits, we evaluated the levels of proteins 14-3-3 and tau in the CSF samples of 140 Chinese patients of 14 different types of gPrDs.

**Results:**

We found that CSF 14-3-3 ELISA values in the patients with P102L GSS and D178N FFI were remarkably low, while those in the patients with T188K, E196A, and E200K gCJD were relatively high. Linear correlation assays identified a positive correlation between positive rate in Western blot (WB) and ELISA values of CSF 14-3-3. ELISA assays for total tau in CSF samples identified relatively high levels in the cases of T188K, E196A, and E200K gCJD (median: 133840.81, 159992.80, and 153342.92 AU/ml), but relatively low levels in those of P102L GSS and D178N FFI (median: 64397.77 and 43856.79 AU/ml).

**Conclusion:**

These data illustrate heterogeneous profiles of CSF 14-3-3 and tau in various types of gPrDs, depending on the differences in the mutations in *PRNP*.

## Introduction

Human genetic prion diseases (gPrDs) account for approximately 10–15% in all human prion diseases worldwide (Prusiner, 1998) and consist of several clinical subtypes, such as genetic Creutzfeldt-Jakob disease (CJD), Gerstmann–Sträussler-Scheinker syndrome (GSS) and fatal familial insomnia (FFI). More than 50 different mutations in the gene encoding the prion protein (*PRNP*) have been noted to be directly associated with gPrDs (Kovacs et al., 2005; Mead, 2006; Mastrianni, 2010). Largely due to the positions of the mutations, different gPrDs may exhibit different clinical, pathogenic and neuropathological characteristics. The features of clinical examinations including electroencephalography (EEG) and MRI, and laboratory tests for some proteins in cerebrospinal fluid (CSF), also vary markedly (Gambetti et al., 2003; Knight et al., 2004; Mead, 2006; Ladogana et al., 2009).

In the past two decades, screening biomarker in CSF for diagnosis of human prion disease has constantly attracted great attention. Several surrogate biomarkers in CSF have been proposed (Van Everbroeck et al., 2005; Ladogana et al., 2009; Pennington et al., 2009). However, the identification of protein 14-3-3 in CSF with Western blot (WB) is almost the only candidate that is accepted worldwide and included in the diagnostic criteria for sporadic CJD (sCJD) (World Health Organization [WHO], 2003). Besides CSF 14-3-3 in WB and positive RT-QuIC in CSF and other tissues for PrP pathological seeding activity, the protein tau in CSF, usually measured by ELISA, is another potent biomarker for diagnosis for human prion diseases, particularly for sCJD (Matsui et al., 2011; Leitao et al., 2016; Humpel and Benke, 2017). Although lots of studies have been conducted to evaluate the potential CSF 14-3-3 and tau in diagnosis for human gPrDs, the significance is still unsettled. Additionally, the profiles of CSF 14-3-3 and tau in various types of genetic PrDs, especially in Chinese patients, needs to be further explored.

In the present study, we tested 140 CSF specimens from the Chinese patients of gPrDs covering 14 different subtypes of mutations in *PRNP*. Among them, the case numbers of D178N FFI, T188K gCJD, E200K gCJD, P105L GSS, and E196A gCJD were top five. Besides routine 14-3-3 specific WBs, all CSF samples were subjected to the ELISA tests for 14-3-3 and tau with commercial kits. In addition, the possible associations of the elevations of the ELISA values of CSF 14-3-3 and tau with some main clinical and laboratory indexes were statistically analyzed.

## Materials and Methods

### CSF Samples and Data Collection

**FIGURE 1:**
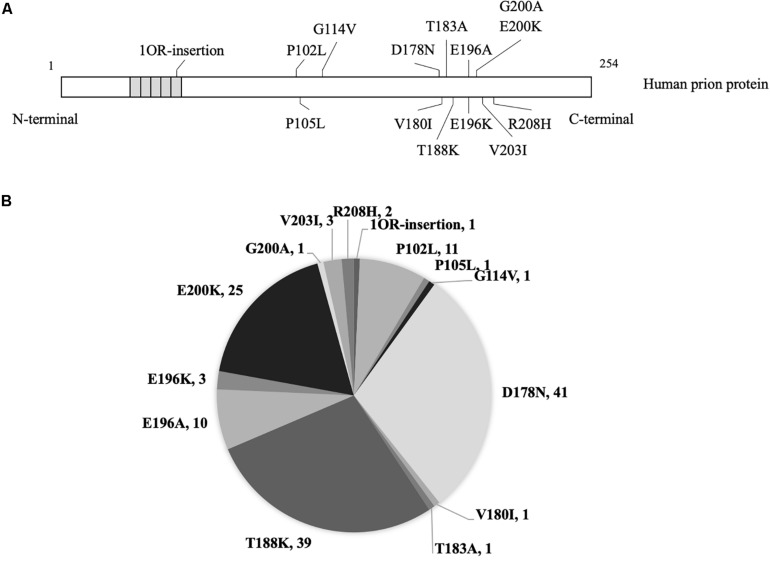
The mutants and numbers of enrolled patients with genetic prion disease. **(A)** Mutations of prion protein in 140 cases with genetic prion disease. **(B)** Number of various genetic prion disease cases in the present study.

A total of 140 CSF samples from the patients with various gPrDs were obtained from the CSF bank in the Center of Chinese CJD Surveillance System (Figure 1). The demography information of the patients, the clinical data, MRI and EEG data, the results of the WB for CSF 14-3-3, and *PRNP* sequencing were collected from the database of the Center of Chinese CJD Surveillance System.

All enrolled CSF samples were obtained by standard clinical procedures and were free of blood contamination. Routine CSF biochemistry assays of those specimens, including cell count, glucose and total protein were all in the normal ranges.

### ELISA for Protein 14-3-3 in CSF

Besides the WB for CSF 14-3-3, the protein 14-3-3 in CSF of each patient was measured by a commercial double-antibody sandwich ELISA and CSF samples were diluted by 40-fold dilution with dilution buffer according to the manufacturer’s instructions (CY8082, CircuLex, Japan). Briefly, 2.5 μl of CSF sample together with 97.5 μl of sample dilutions were transferred into a 96-well 14-3-3 Gamma ELISA kit and incubated at room temperature (RT) for 60 min. After being thoroughly washed with washing buffer four times, the solution of 14-3-3 Gamma detection antibody was added and incubated at RT for 60 min. After being washed, the solution containing HRP-conjugated antibody was added and incubated at RT for 60 min. The reactions were developed with addition of the solution of substrate for 15 min and terminated with the stop solution. Each reaction was measured automatically at 450 nm in an ELISA reader (PerkinElmer, United States). The values of CSF 14-3-3 were correlated to the external standard curve supplied by the manufacturer and measured in arbitrary unit (AU) per ml. Three CSF samples (one P102L, one D178N, and one E196K) showed the negative reaction, whose data were under the threshold of the standard curve range and were set as 0 in the following calculation.

### ELISA for Protein Tau in CSF

The values of total tau protein in CSF were quantitatively measured with a commercial ELISA kit (81572, Innotest hTau-Ag, Belgium). Briefly, 25 μl of CSF sample was diluted together with dilution buffer supplied by the manufacturer and added to duplicate wells of the antibody-coated plate. Subsequently, the plate was incubated overnight at RT. After being washed well five times, 100 μl of HRP-conjugated detection antibodies were added into each well and incubated at RT for 30 min. The reactions were developed with an addition of 100 μl substrate working solution for 30 min in the dark, and terminated with the stop solution. Absorbance at 450 nm was measured by a microplate reader (PerkinElmer, United States) after terminating the reaction by addition of 2 M H_2_SO_4_. CSF tau concentrations were calculated based on a tau standard curve.

### Statistical Analysis

The data were processed with SPSS 17.0 statistics software, and descriptive data were expressed as median (range) for continuous variables and as percent (%) for categorical variables. The Mann–Whitney *U* test was performed for statistical analysis among the groups of positive and negative data in 14-3-3 WB and the categorical variables were compared using the Chi-square test. Multivariate logistic regression was used to analyze associations of CSF 14-3-3 or tau with relational influence factors. The *p*-values were corrected by the Bonferroni’s correction method and presented as ^∗∗∗^ (*p* < 0.001), ^∗∗^ (*p* < 0.01), ^∗^ (*p* < 0.05) and ns (non-significant) on the graphs.

## Results

In total, 140 Chinese patients of various gPrDs were enrolled in this study. All cases were verified to contain a special disease-associated mutation in *PRNP* by sequencing. As shown in Figure 1, 41 were D178N FFI (29.29%), 39 were T188K gCJD (27.86%), 25 were E200K gCJD (17.86%), 11 were P102L GSS (7.86%), 10 were E196A gCJD (7.14%), 3 were E196K gCJD (2.14%), 3 were V203I gCJD (2.14%), and 2 were R208I gCJD (1.43%). There was only one case with the mutation of P105L, G114V, V180I, T183A, G200A or with 1 octarepeat (OR) insertion, respectively. Except for one E200K-129M/V and one T188K-219E/K case, the other 138 cases of gPrDs were methionine/methionine (M/M) homozygous at codon 129 and glutamic acid (E/E) at codon 219.

### The ELISA Values of CSF 14-3-3 in the Patients With Various gPrDs

**FIGURE 2:**
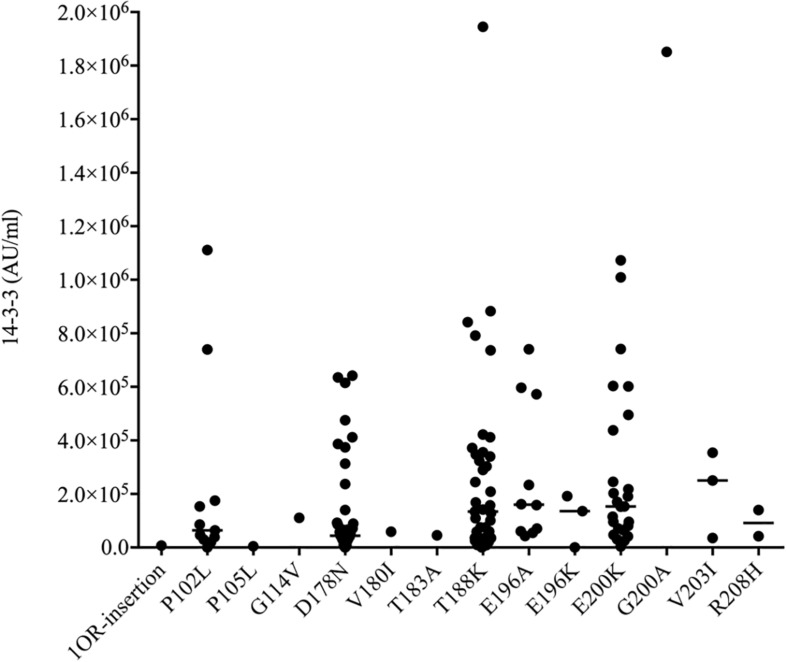
Scatter graph of CSF 14-3-3 levels in 140 patients with various mutants of prion protein. The concentrations of CSF 14-3-3 (AU/ml) were shown in *Y*-axis. Various mutants were shown under each group. Solid line among each group presents the median of the data.

Using a commercial 14-3-3 gamma detection ELISA kit, the values of CSF 14-3-3 in 140 patients of different gPrDs were measured. The ELISA values of CSF 14-3-3 in 140 CSF samples ranged widely from 0 to 1944890.83 AU/ml (Supplementary Table S1). The median values of CSF 14-3-3 were calculated based on the mutant groups and illustrated in Figure 2. In the mutant groups containing more than 10 cases, the median values of CSF 14-3-3 were 133840.81, 159992.80, and 153342.92 AU/ml in the patients of T188K, E196A, and E200K gCJD, respectively, while that of patients of P102L GSS and D178N FFI were 64397.77 and 43856.79 AU/ml, respectively, showing significantly lower CSF 14-3-3 levels compared with those of T188K, E196A, and E200K gCJD cases (*p* < 0.01). It implies that P102L GSS and D178N FFI cases have different homeostasis of CSF 14-3-3 compared with the other genetic prion mutants.

### Correlation of CSF 14-3-3 in gPrDs Was Detected by Western Blot and ELISA

**FIGURE 3:**
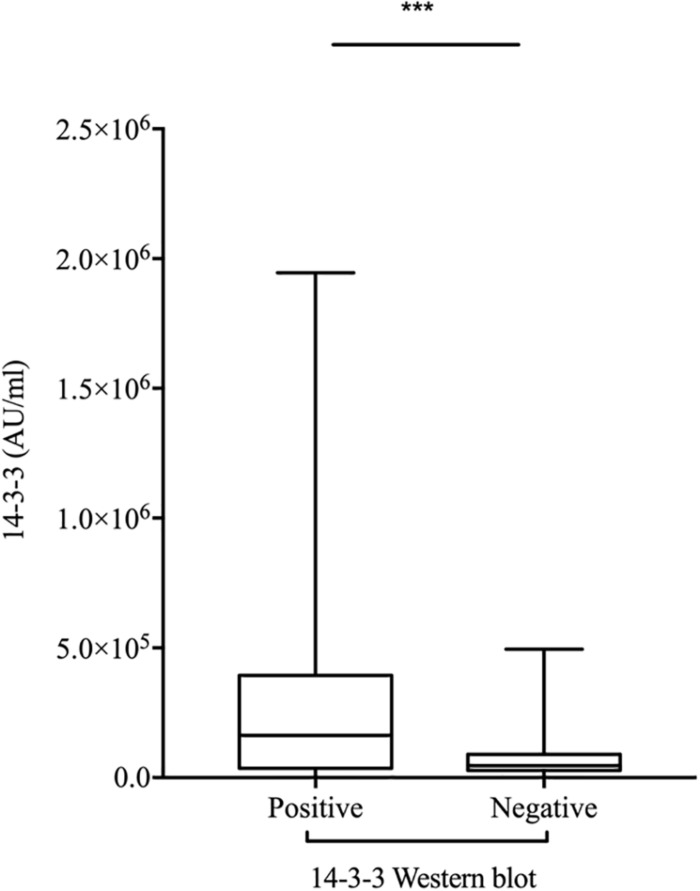
Comparison of the two methods for CSF 14-3-3 detection: ELISA vs. WB. ELISA 14-3-3 levels in CSF of patients with various *PRNP* mutants according to the 14-3-3 WB results (Positive and Negative). The solid line represents the median in each group. ^∗∗∗^On behalf of *p* < 0.001.

To evaluate the relationship of ELISA values of CSF 14-3-3 with the results of WB of CSF 14-3-3, the tested samples were classified into WB-positive and WB-negative groups according to the previous data of WBs. Figure 3 revealed remarkably higher CSF 14-3-3 ELISA values in WB-positive group (median: 180892.03 AU/ml) than those of WB-negative group (median: 45883.95 AU/ml), with significant statistical differences (*p* < 0.0001). Only the 20.7% of WB-negative samples were in the groups of high ELISA values (Table 1).

**TABLE 1:** Percentages of CSF samples in the group of positive and negative of 14-3-3 Western blot according to various 14-3-3 value ranges.

**14-3-3 Western blot**	**14-3-3% (Case no./Group no.)**			
	**<10000**	**10000 < 100000**	**100000–500000**	**>500000**
	**AU/ml**	**AU/ml**	**AU/ml**	**AU/ml**
Positive	8.5 (7/82)	26.8 (22/82)	41.5 (34/82)	23.2 (19/82)
Negative	13.8 (8/58)	65.5 (38/58)	20.7 (12/58)	0 (0/58)

**FIGURE 4:**
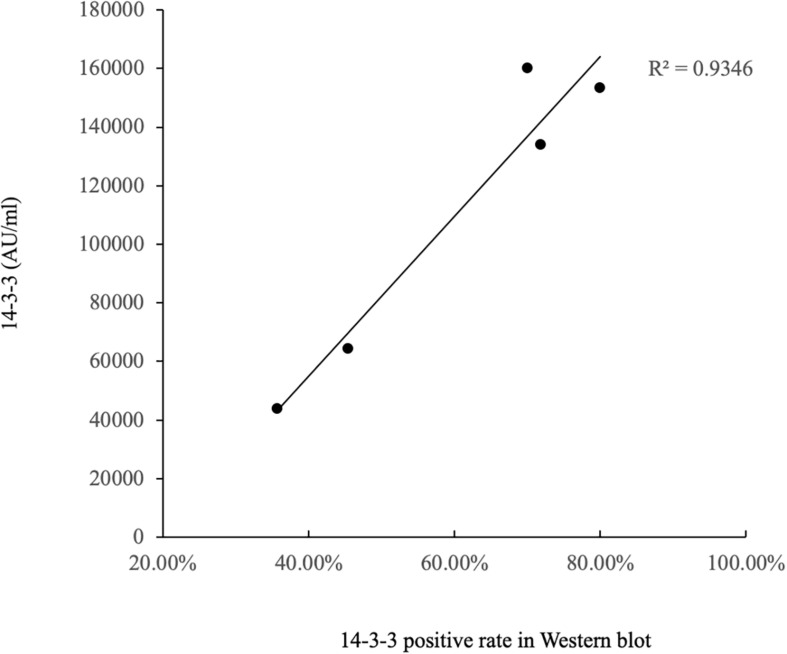
Correlation analysis of WB-positive rate and ELISA values in CSF 14-3-3 of patients of various gPrDs. The concentrations of CSF 14-3-3 (pg/ml) are showed in *Y*-axis. Solid line presents the trend among the groups.

The ELISA values of CSF 14-3-3 of the tested samples were further grouped based on the disease associated *PRNP* mutations (Table 2). Comparing the ELISA values of CSF 14-3-3 in each *PRNP* mutation, there was no statistical difference between the WB-positive and negative groups of P102L GSS, E196A, and E200K gCJD that contained more than 10 cases, although the medians of ELISA values in the WB-positive group were higher than those of WB-negative groups. However, the ELISA values of CSF 14-3-3 in the WB-positive groups of D178N FFI were significantly higher than that of the individual WB-negative groups (*p* < 0.01). Subsequently, the WB-positive rate and ELISA values of CSF 14-3-3 in each group that contained more than 10 cases were separately analyzed. The WB-positive rate of D178N FFI, P102L GSS, E196A, T188K, and E200K gCJD were 36.59, 45.45, 70, 71.79, and 80%, while the corresponding ELISA median values were 43856.79, 64397.77, 159992.80, 133840.81, and 153342.92 AU/ml, respectively. Linear correlation assays identified a positive correlation between WB-positive rate and ELISA values of CSF 14-3-3 (*R*^2^ = 0.9346, Figure 4). Taken together, these results highlight a close association between two techniques.

**TABLE 2:** Comparison of CSF 14-3-3 level in positive vs. negative group of 14-3-3 detected by WB in 140 cases with various prion protein mutants.

**Mutants**	**Case no.**	**14-3-3 Western blot**					
		**Positive**		**Negative**		***p*-value^∗^**
		**Case no.**	**Median (min, max)**	**Case no.**	**Median (min, max)**	
1OR-insertion	1	1	7190.38	0	–	–
P102L	11	5	85487.75 (29843.13, 1110947.75)	6	55383.23 (0, 175329.80)	0.429
P105L	1	0	–	1	4331.97	–
G114V	1	0	–	1	110925.92	–
D178N	41	15	312947.34 (2190.54, 642323.70)	26	40703.11 (0, 237548.28)	0.007
V180I	1	0	–	1	59076.23	–
T183A	1	0	–	1	45593.27	–
T188K	39	28	200908.8 (728.20, 1944890.83)	11	59889.00 (8587.07, 347672.03)	0.095
E196A	10	7	234195.73 (54644.75, 740488.50)	3	71071.80 (42897.03, 161699.20)	0.267
E196K	3	2	163988.92 (135827.78, 192150.05)	1	0.00	0.667
E200K	25	20	161908.79 (3749.00, 1072551.49)	5	95731.73 (46951.74, 495221.73)	0.668
G200A	1	1	1851829.29	0	–	–
V203I	3	2	302406.26 (250852.98, 353959.53)	1	35348.51	0.667
R208H	2	1	140331.47	1	42322.88	–

*^∗^Mann–Whitney *U* test.*

### Total Tau Profiles in CSF of Patients With Various gPrDs

**FIGURE 5:**
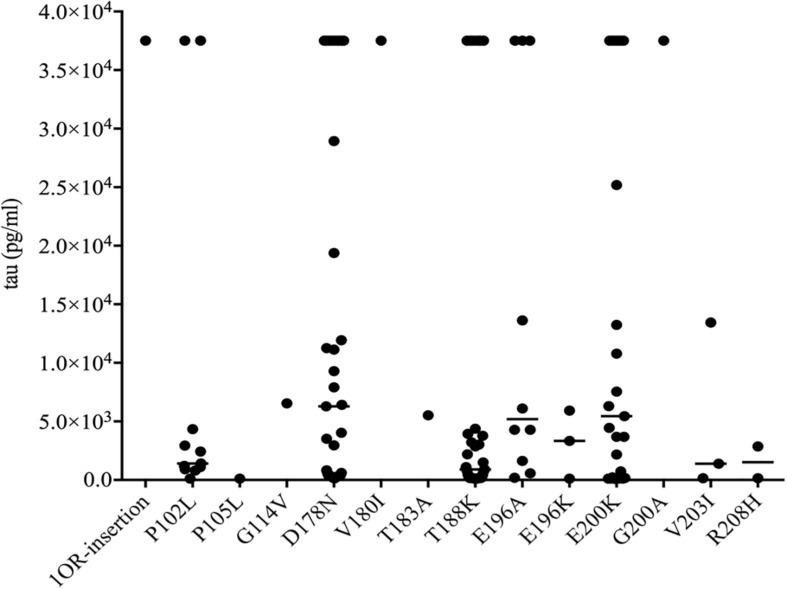
Scatter graph of tau levels in CSF of the patients with various mutants of prion protein. The concentrations of CSF tau (pg/ml) were shown in *Y*-axis. Various mutants were shown under each group. Solid line among each group presents the median of the data.

To see the tau levels of CSF samples from patients with various gPrDs, the CSF samples from 140 *PRNP* mutant cases were subjected to a tau ELISA with the commercial total hTau kit. As shown in Figure 5, the CSF tau values varied largely among the tested samples, even in the cases with the same disease associated *PRNP* mutation (Supplementary Table S1). About a quarter of patients (36/140) had CSF tau values above the upper limit of the standard range and these data were set as the maximum of the standard curve (37517.83 pg/ml) according to the dilution ratio. However, the distributions of CSF tau values revealed the disease-associated patterns. In five disease groups containing more than 10 cases, the medians of CSF tau values in the patients of D178N FFI (6285.30 pg/ml), E196A (5198.53 pg/ml) and E200K gCJD (5447.27 pg/ml) were high, while those of P102L GSS (1404.33 pg/ml) and T188K gCJD (899.97 pg/ml) were low, showing significant differences (*p* < 0.01, Figure 5). Furthermore, to better understand the CSF tau levels distribution, we divided the tau values of all CSF samples into four groups, and it showed that the tau values in 54.55% (6/11) of P102L GSS and 56.41% (22/39) of T188K gCJD were below 2000 pg/ml, while 41.47% (17/41) of D178N FFI, 30% (3/10) of E196A gCJD and 32.0% (8/25) of E200K gCJD were in this range. On the other hand, only 18.18% (2/11) of P102L GSS and 25.64% (10/39) of T188K gCJD revealed high tau values (above 10000 pg/ml), but 43.9% (18/41) of FFI, 40% (4/10) of E196A gCJD and 40.0% (10/25) of E200K gCJD were in this zone (Table 3). It seems that relatively high portions of the Chinese patients of P102L GSS and T188K gCJD have low CSF tau levels, while high portions of the cases of D178N FFI, E196A gCJD, and E200K gCJD show high CSF tau levels.

**TABLE 3:** Percentages of CSF samples among different human genetic prion diseases according to various total tau value ranges.

**Groups**	**Tau % (Case no./Group no.)**			
	**<2000**	**2000 < 10000**	**10000–30000**	**>30000**
	**pg/ml**	**pg/ml**	**pg/ml**	**pg/ml**
P102L GSS	54.55 (6/11)	27.27 (3/11)	0.0 (0/11)	18.18 (2/11)
D178N FFI	41.47 (17/41)	14.63 (6/41)	14.63 (6/41)	29.27 (12/41)
T188K gCJD	56.41 (22/39)	17.95 (7/39)	0.0 (0/39)	25.64 (10/39)
E200K gCJD	32.0 (8/25)	28.0 (7/25)	12.0 (3/25)	28.0 (7/25)
E196A gCJD	30 (3/10)	30 (3/10)	10 (1/10)	30 (3/10)

### Relationship of CSF 14-3-3 and Total Tau With Other Potential Factors

To identify the potential influence factors correlated with the CSF 14-3-3 and tau levels, some clinical and laboratory indexes from the enrolled patients were selected and statistically analyzed. Multivariate linear regression analysis showed a significant correlation of the positive of CSF 14-3-3 in WB with the elevations of both CSF 14-3-3 and tau (*p* < 0.0001). Additionally, one parameter of clinical auxiliary examinations, sCJD-associated abnormal changes in MRI, showed remarkable correlations with the elevation of CSF 14-3-3 (*p* < 0.05). Meanwhile, the patients with relative short disease duration showed association with a high level of CSF tau (*p* < 0.05, Table 4).

**TABLE 4:** Multivariate linear regression analysis of the influence of CSF 14-3-3 and tau levels with related influence factors.

**Factors**	***p*-value**	
	**CSF 14-3-3**	**CSF tau**
Gender	0.350	0.380
Age	0.139	0.349
14-3-3 Western blot	<0.0001	<0.0001
PSWC in EEG	0.277	0.314
MRI abnormal changes	0.015	0.175
Rapid progressive dementia	0.077	0.313
Myoclonus	0.113	0.380
Visual or cerebellar disturbance	0.052	0.097
Pyramidal or extrapyramidal dysfunction	0.051	0.101
Akinetic mutism	0.487	0.058
Disease duration	0.174	0.038

Further, the above variables were introduced into the multivariate linear regression analyses of some subtypes of gPrDs, including D178N FFI, T188K and E200K gCJD. As summarized in Table 5, positive CSF 14-3-3 in WB illustrated remarkable correlation with the elevations of CSF 14-3-3 and tau in D178N FFI cases, but not in T188K and E200K cases. D178N FFI patients with myoclonus tended to have relative high CSF tau levels. T188K gCJD patients with relative short disease duration were prone to have relative high CSF tau levels. E200K gCJD patients who showed PSWC (periodic sharp wave complexes) in EEG seemed to positively associate with the elevated CSF tau levels. The rest of the tested elements did not reveal any correlation with the elevations of CSF 14-3-3 and tau values in ELISA.

**TABLE 5:** Multivariate linear regression analysis of the influence of CSF 14-3-3 and tau levels with related influence factors in various prion protein mutants.

**Factors**	**D178N (*p*-value)**		**T188K (*p*-value)**		**E200K (*p*-value)**	
	**CSF 14-3-3**	**CSF tau**	**CSF 14-3-3**	**CSF tau**	**CSF 14-3-3**	**CSF tau**
Gender	0.231	0.136	0.124	0.241	0.313	0.272
Age	0.279	0.484	0.203	0.497	0.187	0.235
14-3-3 Western blot	0.001	<0.0001	0.052	0.148	0.229	0.297
PSWC in EEG	0.213	0.430	0.297	0.311	0.228	0.025
MRI abnormal changes	0.174	0.389	0.344	0.200	0.216	0.454
Rapid progressive dementia	0.402	0.154	0.289	0.139	0.210	0.067
Myoclonus	0.204	0.253	0.157	0.325	0.262	0.424
Visual or cerebellar disturbance	0.302	0.137	0.256	0.099	0.244	0.229
Pyramidal or extrapyramidal dysfunction	0.155	0.480	0.261	0.233	0.130	0.141
Akinetic mutism	0.170	0.276	0.341	0.217	0.456	0.159
Disease duration	0.247	0.207	0.171	0.038	0.269	0.460

## Discussion

In this study, we have evaluated the CSF levels of protein 14-3-3 and tau in 140 Chinese patients with various gPrDs using commercial ELISA kits. Although CSF 14-3-3 can be detected in most of the cases (97.9%, 137/140), the concentrations vary dramatically, even in the same types of gPrD. Generally, the cases with the mutations in the N-terminal part of PrP (before aa 183) seem to contain lower CSF 14-3-3 levels than those having mutations in the C-terminal part of PrP, despite that many mutants enrolled in the present study contain only a few cases. Among 14 different types of gPrDs in this study, 5 have 10 or more cases. As expected, the median ELISA values of CSF 14-3-3 in the groups of T188K, E196A, and E200K gCJD cases are higher than that of P102L GSS and D178N FFI cases. From the aspect of clinical manifestations, T188K, E196A, and E200K gCJD are more like sCJD (Shi et al., 2009, 2015, 2016, 2017; Gao et al., 2010; Chen et al., 2013; Zhang et al., 2014). In CSF laboratory, higher ratios of T188K, E196A, and E200K gCJD cases reveal 14-3-3 positive in WB. Additionally, we previously reported that the Chinese patients with T188K, E196A, and E200K gCJD have relatively shorter average disease durations than that of P102L GSS and D178N FFI (Shi et al., 2015). As abnormal appearance of 14-3-3 in CSF reflects brain damage, high CSF 14-3-3 levels in the patients of prion diseases might associate with the rapid and massive neuronal damage in CNS. Similar as the data reported worldwide (Kovacs et al., 2005; Nozaki et al., 2010; Jeong and Kim, 2014), Chinese patients of P102L GSS and D178N FFI show relatively slow progression and a longer survival time than those of sCJD, as well as of T188K and E200K gCJD (Shi et al., 2015, 2018). The profiles of CSF 14-3-3 in different gPrDs may reflect their clinical features.

Detection of CSF 14-3-3 protein by 14-3-3-specific WB is widely used as a diagnostic criteria for probable sCJD (World Health Organization [WHO], 2003; Parchi and Capellari, 2013). Some studies have already evaluated the ELISA method of CSF 14-3-3 in the patients with sCJD and verified very good accuracy (Matsui et al., 2011; Leitao et al., 2016; Schmitz et al., 2016; Humpel and Benke, 2017). Good consistency between the CSF 14-3-3 concentration in ELISA and 14-3-3 positive in WB has also proposed in the gPrD cases in this study. The median ELISA value of CSF 14-3-3 in WB-positive group is about 4 times higher than those in WB-negative group. However, there are several cases revealing discrepant reactivities in those two methods. One possible explanation is the difference in target antigen of two methodologies, since WB mainly recognizes the linear epitope while ELISA favors special epitope. Differences in the antibodies used in those two tests may also be a factor, since the antibody in WB is a pan 14-3-3 that can recognize all subtypes of 14-3-3, while that in ELISA recognizes 14-3-3 gamma isoform.

The increase of CSF total tau has been repeatedly observed in the patients affected by sCJD, and it is considered to be another good diagnostic biomarker for sCJD (Ladogana et al., 2009; Zerr et al., 2017). The alterations of CSF tau in various gPrDs are also described (Sanchez-Juan et al., 2006; Chen et al., 2010; Llorens et al., 2016). Ladogana et al. (2009) have conducted a cohort study containing 174 patients with various gPrDs, revealing relatively low total tau level in GSS and FFI patients, but high tau level in the other gCJD types with the cutoff value of 1300 pg/ml. Consistent with these studies, our data here also show relatively high median values of CSF total tau in E200K and E196A gCJD cases, and low value in P102L GSS cases. However, tau values in Chinese D178N FFI patients seem not to be consistent with the observations described in previous reports, as the CSF tau amounts in roughly 60% tested D178N FFI cases are above 2000 pg/ml and about 44% cases are above 10000 pg/ml. On the other hand, T188K gCJD, which is the second most commonly observed gPrD in Chinese but rarely described in the other countries, shows very low median of CSF tau. Different types of gPrDs look to have different profiles of CSF tau.

We have analyzed the potential factors that may contribute to the increases of CSF 14-3-3 and tau measured by ELISA in gPrDs. Similar as the positive CSF 14-3-3 in WB, no special clinical element is closely associated with the elevation of 14-3-3 in CSF, besides MRI abnormality and PSWC in EEG. CSF 14-3-3 positive in WB is the most significant factor responsible for elevation of CSF tau in all gPrDs, which may somehow reflect the abnormality in CSF homeostasis (Ladogana et al., 2009; Llorens et al., 2016). Analysis has also proposed a few influence factors for CSF tau levels, such as myoclonus in D178N FFI and disease duration in T188K gCJD, but the clinical significances need to be further explored. In addition, some literature has reported that codon 129 genotype in *PRNP* influences the levels of CSF 14-3-3 and tau in gPrD cases (Ladogana et al., 2009). However, the polymorphisms of codon 129 of the Chinese cases of gPrDs in this study are almost Met/Met homozygote, except one Met/Val one. Therefore, it is impossible to see the potential effect of polymorphisms of codon 129 on CSF 14-3-3 and tau among Chinese gPrD cases.

Because the main aim of this study is evaluation of the possible different levels of CSF 14-3-3 and tau in various gPrDs via ELISA method, the diagnostic potential for gPrD is not addressed. Although different gPrDs appear in the distinguishable ELISA profiles of CSF 14-3-3 and tau, the individual concentrations of CSF 14-3-3 and tau vary largely even in the same subtype of gPrD, which may hinder the usage for diagnosis of gPrDs. Certainly, the study here does not contain controls, such as the CSF samples of sCJD, non-CJD and other diagnosed neurodegenerative diseases. It is impossible to figure out the cutoff values in those two ELISA tests for gPrDs. Therefore, the clinical significances of ELISA 14-3-3 and tau in gPrDs need further study.

## Conclusion

These data illustrate heterogeneous profiles of CSF 14-3-3 and tau in various types of gPrDs, highly depending on the differences in the mutations in *PRNP*.

## Data Availability

All datasets generated for this study are included in the manuscript and/or the Supplementary Files.

## Ethics Statement

Usages of stored CSF samples and relevant clinical information of the patients with gPrDs in the Center of Chinese CJD Surveillance System has been approved by the Ethics Committee of the National Institute for Viral Disease Control and Prevention, China CDC.

## Author Contributions

CC and CH designed the study and drafted the manuscript. WZ provided the patients data. CH, JC, and QS carried out the ELISA. KX, YW, LL, and YX performed the statistical analysis. X-PD conceived of the study, participated in the study design and coordination, and helped to draft the manuscript. All authors read and approved the final manuscript.

## Conflict of Interest Statement

The authors declare that the research was conducted in the absence of any commercial or financial relationships that could be construed as a potential conflict of interest.
