# Supplementary material for: Profiles of 14-3-3 and Total Tau in CSF Samples of Chinese Patients of Different Genetic Prion Diseases
Source: Front Neurosci. 2019 Sep 4;13:934. doi: 10.3389/fnins.2019.00934 (PMC6737049; doi:10.3389/fnins.2019.00934)
Supplement: Supplementary file 1 [file Table_1.DOCX]

Supplementary Table 1 ELISA 14-3-3 and tau values in CSF samples of patients with various prion protein mutants

| No. | Mutant | Tau (pg/ml) | 1433 (AU/ml) |
| --- | --- | --- | --- |
| 1 | G200A | 37517.83 | 1851829.288 |
| 2 | T188K | 145.88 | 1944890.827 |
| 3 | 1OR0insertion | 37517.83 | 7190.37668 |
| 4 | P102L | 1207.43 | 153860.8147 |
| 5 | D178N | 3518.03 | 66468.68944 |
| 6 | D178N | 9303.32 | 71915.67 |
| 7 | G114V | 6548.07 | 110925.9189 |
| 8 | D178N | 607.41 | 23248.8766 |
| 9 | D178N | 37517.83 | 7036.73244 |
| 10 | E196K | 3349.52 | 0 |
| 11 | D178N | 37517.83 | 57255.41452 |
| 12 | T188K | 37517.83 | 728.19972 |
| 13 | D178N | 247.81 | 37401.09128 |
| 14 | T188K | 37517.83 | 208391.5818 |
| 15 | T188K | 126.77 | 133840.813 |
| 16 | D178N | 37517.83 | 34051.6864 |
| 17 | T188K | 105.17 | 34606.56668 |
| 18 | P102L | 4339.77 | 64397.76724 |
| 19 | D178N | 11260.55 | 2190.53544 |
| 20 | P102L | 817.51 | 175329.8028 |
| 21 | R208H | 163.27 | 42322.87712 |
| 22 | D178N | 37517.83 | 35348.50488 |
| 23 | T188K | 37517.83 | 20641.18232 |
| 24 | D178N | 7902.97 | 92136.52944 |
| 25 | E196A | 13636.45 | 234195.7323 |
| 26 | V203I | 145.88 | 250852.9838 |
| 27 | T188K | 179.36 | 128422.9523 |
| 28 | E200K | 4448.76 | 41178.427 |
| 29 | E196A | 565.40 | 158286.3999 |
| 30 | T188K | 145.88 | 142095.3115 |
| 31 | D178N | 194.45 | 237548.2765 |
| 32 | E200K | 25180.62 | 170474.6518 |
| 33 | R208H | 2861.81 | 140331.47 |
| 34 | T188K | 179.36 | 356193.424 |
| 35 | D178N | 6285.30 | 27142.90444 |
| 36 | D178N | 37517.83 | 46174.63228 |
| 37 | D178N | 409.35 | 28939.08276 |
| 38 | D178N | 2964.15 | 312947.3392 |
| 39 | V180I | 37517.83 | 59076.23384 |
| 40 | E200K | 10789.09 | 65017.63688 |
| 41 | T188K | 3770.59 | 302933.6018 |
| 42 | D178N | 304.62 | 4185.5254 |
| 43 | E200K | 5447.27 | 24832.13764 |
| 44 | E200K | 126.77 | 95731.72672 |
| 45 | E200K | 37517.83 | 30387.44544 |
| 46 | E200K | 3687.35 | 3749.00424 |
| 47 | E200K | 6316.89 | 1009253.338 |
| 48 | T183A | 5518.21 | 45593.2728 |
| 49 | T188K | 756.66 | 35906.52424 |
| 50 | D178N | 586.61 | 41368.81084 |
| 51 | D178N | 11137.87 | 40608.13696 |
| 52 | T188K | 817.51 | 74460.0794 |
| 53 | D178N | 382.73 | 85927.268 |
| 54 | T188K | 2861.81 | 841725.6914 |
| 55 | D178N | 28934.14 | 139828.794 |
| 56 | D178N | 417.98 | 48317.05164 |
| 57 | T188K | 483.36 | 35906.52424 |
| 58 | D178N | 11929.51 | 40798.08992 |
| 59 | D178N | 6413.77 | 28939.08276 |
| 60 | D178N | 4025.31 | 0 |
| 61 | T188K | 179.36 | 168599.6599 |
| 62 | D178N | 37517.83 | 387124.9244 |
| 63 | E200K | 37517.83 | 603191.1973 |
| 64 | T188K | 899.97 | 8587.07164 |
| 65 | P102L | 126.77 | 38716.55012 |
| 66 | E200K | 105.17 | 202634.09 |
| 67 | D178N | 19380.42 | 55044.89276 |
| 68 | T188K | 1108.52 | 412216.6073 |
| 69 | E200K | 2173.62 | 153342.9242 |
| 70 | P102L | 1097.47 | 0 |
| 71 | T188K | 37517.83 | 58670.65988 |
| 72 | E200K | 126.77 | 80905.0044 |
| 73 | T188K | 163.27 | 10006.97832 |
| 74 | T188K | 37517.83 | 883018.0384 |
| 75 | D178N | 145.88 | 43856.79224 |
| 76 | E200K | 179.36 | 741718.3913 |
| 77 | E196K | 126.77 | 135827.776 |
| 78 | D178N | 37517.83 | 412216.6073 |
| 79 | E196A | 37517.83 | 596933.2929 |
| 80 | T188K | 208.72 | 290058.9904 |
| 81 | T188K | 1503.56 | 791677.8974 |
| 82 | T188K | 179.36 | 421612.0428 |
| 83 | T188K | 37517.83 | 6425.44408 |
| 84 | T188K | 2199.73 | 59889.00332 |
| 85 | E200K | 126.77 | 245244.6604 |
| 86 | E200K | 37517.83 | 152825.6143 |
| 87 | T188K | 145.88 | 244315.412 |
| 88 | T188K | 126.77 | 157502.349 |
| 89 | P102L | 911.54 | 85487.75168 |
| 90 | T188K | 459.52 | 25185.82452 |
| 91 | D178N | 409.35 | 30205.8514 |
| 92 | T188K | 145.88 | 372049.9846 |
| 93 | E200K | 3687.35 | 191309.4117 |
| 94 | D178N | 163.27 | 10325.44828 |
| 95 | D178N | 259.87 | 41178.427 |
| 96 | T188K | 271.55 | 37026.56468 |
| 97 | E196A | 37517.83 | 740488.5021 |
| 98 | E196A | 37517.83 | 572376.5099 |
| 99 | V203I | 1387.88 | 35348.50488 |
| 100 | E196A | 4279.55 | 60296.19812 |
| 101 | E200K | 126.77 | 46951.73956 |
| 102 | D178N | 37517.83 | 373963.5634 |
| 103 | P105L | 126.77 | 4331.97196 |
| 104 | E200K | 13245.46 | 217737.8093 |
| 105 | D178N | 37517.83 | 475438.0996 |
| 106 | T188K | 126.77 | 7653.21004 |
| 107 | T188K | 3034.78 | 100502.5232 |
| 108 | E200K | 731.75 | 95731.72672 |
| 109 | P102L | 2938.17 | 46368.69916 |
| 110 | T188K | 3211.04 | 74886.01292 |
| 111 | E200K | 37517.83 | 1072551.494 |
| 112 | V203I | 13437.28 | 353959.5283 |
| 113 | P102L | 37517.83 | 1110947.754 |
| 114 | D178N | 37517.83 | 642323.7024 |
| 115 | D178N | 823.49 | 89240.64256 |
| 116 | T188K | 4370.43 | 109286.483 |
| 117 | E196K | 5930.81 | 192150.0528 |
| 118 | E200K | 37517.83 | 602144.8601 |
| 119 | P102L | 1404.33 | 19266.85472 |
| 120 | E200K | 37517.83 | 495221.7266 |
| 121 | E196A | 1620.89 | 161699.2049 |
| 122 | D178N | 335.36 | 29300.22964 |
| 123 | E200K | 37517.83 | 438261.0376 |
| 124 | D178N | 345.18 | 4773.97376 |
| 125 | E200K | 222.30 | 70861.16488 |
| 126 | T188K | 37517.83 | 736197.0892 |
| 127 | D178N | 37517.83 | 635218.3415 |
| 128 | T188K | 3946.08 | 41750.00836 |
| 129 | E196A | 194.45 | 42897.0294 |
| 130 | T188K | 37517.83 | 323836.0709 |
| 131 | P102L | 37517.83 | 739874.1873 |
| 132 | P102L | 2424.38 | 29843.13208 |
| 133 | E196A | 6102.60 | 54644.74896 |
| 134 | D178N | 282.88 | 66468.68944 |
| 135 | T188K | 37517.83 | 339626.153 |
| 136 | E196A | 4294.47 | 71071.79916 |
| 137 | D178N | 37517.83 | 615321.4704 |
| 138 | E200K | 7554.02 | 114698.264 |
| 139 | T188K | 37517.83 | 347672.0261 |
| 140 | D178N | 293.89 | 3894.02928 |
